# Mouse Pachytene Checkpoint 2 *(Trip13)* Is Required for Completing Meiotic Recombination but Not Synapsis

**DOI:** 10.1371/journal.pgen.0030130

**Published:** 2007-08-10

**Authors:** Xin Li, John C Schimenti

**Affiliations:** Cornell University, College of Veterinary Medicine, Department of Biomedical Sciences, Ithaca, New York, United States of America; National Cancer Institute, United States of America

## Abstract

In mammalian meiosis, homologous chromosome synapsis is coupled with recombination. As in most eukaryotes, mammalian meiocytes have checkpoints that monitor the fidelity of these processes. We report that the mouse ortholog *(Trip13)* of pachytene checkpoint 2 *(PCH2),* an essential component of the synapsis checkpoint in Saccharomyces cerevisiae and *Caenorhabditis elegans,* is required for completion of meiosis in both sexes. TRIP13-deficient mice exhibit spermatocyte death in pachynema and loss of oocytes around birth. The chromosomes of mutant spermatocytes synapse fully, yet retain several markers of recombination intermediates, including RAD51, BLM, and RPA. These chromosomes also exhibited the chiasmata markers MLH1 and MLH3, and okadaic acid treatment of mutant spermatocytes caused progression to metaphase I with bivalent chromosomes. Double mutant analysis demonstrated that the recombination and synapsis genes *Spo11, Mei1, Rec8,* and *Dmc1* are all epistatic to *Trip13,* suggesting that TRIP13 does not have meiotic checkpoint function in mice. Our data indicate that TRIP13 is required after strand invasion for completing a subset of recombination events, but possibly not those destined to be crossovers. To our knowledge, this is the first model to separate recombination defects from asynapsis in mammalian meiosis, and provides the first evidence that unrepaired DNA damage alone can trigger the pachytene checkpoint response in mice.

## Author Summary

It is critical that the chromosomes carried by sperm and eggs contain faithful representations of the genome of the individual that produced them. During the process of meiosis, the maternal and paternal copies of each chromosome “synapse” with each other (become tightly associated), exchange genetic material via the process of recombination, then separate into daughter cells in the first of two meiotic cell divisions. The intricate chromosome behavior is subject to errors, so most organisms have evolved meiotic “checkpoints” that monitor fidelity of chromosome synapsis and repair of DNA damage. These checkpoints cause defective cells to self destruct rather than generate defective sperm or eggs. We studied the effects of deleting mouse *Trip13*, a gene that in distant organisms plays a key role in meiotic checkpoint control. These experiments revealed that instead of having a checkpoint role, *Trip13* is required for one of the two major classes of recombination in meiosis that is required for repairing broken DNA molecules. The chromosomes still synapsed normally, but animals were sterile due to massive death of oocytes and spermatocytes. These results indicate that, in addition to a checkpoint that responds to failed synapsis, one exists to specifically detect unrepaired DNA damage that is due to failed recombination.

## Introduction

The genesis of gametes containing an intact, haploid genome is critical for the prevention of birth defects, and is highly dependent upon the fidelity of chromosome dynamics before the first meiotic division. Homologous chromosomes must pair, synapse, undergo recombination, and segregate properly to opposite poles. Recombination, which repairs repair double strand breaks (DSBs) that are genetically induced in leptonema, is coupled with synapsis in budding yeast and mammals. While our knowledge of the assembly and nature of recombination machinery is extensive, little is known about the disassembly of recombination intermediates, recruitment of DNA replication machinery during recombinational repair, and how the choice between different repair pathways is made.

Defects in recombination can preclude homologous chromosome pairing, leave unrepaired chromosome breaks, and cause aneuploidy by abrogating crossing over. To avoid such deleterious outcomes, surveillance systems (“checkpoints”) exist to sense meiotic errors and eliminate cells containing unresolved defects. In many organisms, including *S. cerevisiae, Drosophila melanogaster, C. elegans,* and mice [1–4], meiocytes with defects in recombination and/or chromosome synapsis trigger meiotic arrest in the pachytene stage of meiotic prophase I. This response to meiotic defects is referred to as the “pachytene checkpoint” (reviewed in [5]). Genetic experiments in S. cerevisiae have identified elements of the pachytene checkpoint machinery (reviewed in [5]). In addition to meiosis-specific proteins, these include factors that play roles in DNA damage signaling in mitotic cells [6–10]. Arabidopsis thaliana does not appear to have a pachytene checkpoint akin to that in yeast [11], nor do male *Drosophila*.

The pachytene checkpoint is known to monitor two aspects of meiotic chromosome metabolism in S. cerevisiae and C. elegans: (1) DSB repair and (2) chromosome synapsis [2,12]. In mice, both spermatocytes and oocytes harboring mutations that disrupt DSB repair (such as *Dmc1, Msh5,* and *Atm*) are efficiently eliminated in pachynema, but spermatocytes are much more sensitive to DSB repair–independent synapsis defects than oocytes [13–15]. However, because recombination is required for synapsis in mice (mutations in recombination genes such as *Dmc1* cause extensive asynapsis [16]), it has remained formally uncertain whether there is a distinct pachytene checkpoint that responds to defects in meiotic recombination, and if so, whether it would be identical to that used in somatic cells. The mechanisms of putative pachytene checkpoint control remain unknown in mammals, since no mutations have been identified that abolish it.

*PCH2,* encoding a nucleolar-localized AAA-ATPase that was originally identified in an S. cerevisiae genetic screen for mutants that relieve pachytene arrest of asynaptic *zip1* mutants [8], was recently determined to be an essential component of the pachytene synapsis (but not DSB repair) checkpoint in yeast and worms [2,12]. *PCH2* orthologs are present in organisms that undergo synaptic meiosis, but not asynaptic meiosis, prompting the suggestion that a Pch2-dependent checkpoint evolved to monitor synaptonemal complex (SC) defects from yeast to humans [12]. Here, we generated mice deficient for the *Trip13,* the ortholog of *PCH2,* and evaluated whether it also plays a role in the pachytene checkpoint. Surprisingly, while we found no evidence for checkpoint function, we did uncover a potential role for this protein in noncrossover (NCO) repair of meiotic DSBs.

## Results

### *Trip13* Is a Widely Expressed Mammalian Ortholog of *PCH2* with Unusual Phylogenetic Relationships

**Figure 1:**
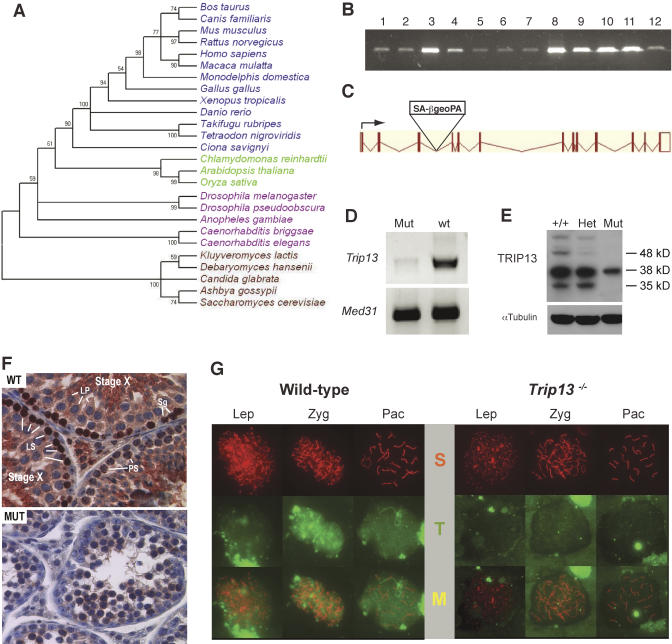
The Mouse PCH2 Ortholog TRIP13 and Expression in Wild Type and Mutant (A) Phylogenetic tree of presumed PCH2/TRIP13 orthologs. The database sequence accession number of each protein is presented in Table S1. Numbers shown are bootstrap values (see Materials and Methods). The clustering was the same regardless of whether the whole entire AA sequence or trimmed sequences (where regions showing little conservation were removed) were used for the analysis. Major eukaryotic groups are indicated in color, with deuterostomia in blue, plants in green, protostomia in purple, and fungi in maroon. (B) Amplification products of cDNA from the following tissues: 1, heart; 2, brain; 3, spleen; 4, lung; 5, liver; 6, skeletal muscle; 7, kidney; 8, testis; 9, E7 embryo; 10, E11 embryo; 11, E15 embryo; and 12, E17 embryo. (C) Intron–exon structure of TRIP13 and insertion site of gene-trap vector. See Materials and Methods for details on how the precise insertion site was identified. (D) RT-PCR of *Trip13* and a control gene Med31 from testis RNA. The *Trip13* primers are situated in the first and last exons (see Materials and Methods). (E) Western blot analysis of testis protein with anti-TRIP13 antibody. The blot was later probed with anti-alpha tubulin actin as a loading control. The expected TRIP13 protein is ∼48 KDa. (F) Localization of TRIP13 in testes. Wild-type (top) and mutant (bottom) testis sections were probed with chicken anti-TRIP13, and detected with HRP-conjugated anti-chicken IgG (brown/red staining). Expression in WT was most prominent in the nuclei of Type B spermatogonia (Sg), leptotene spermatocytes (LS), and early pachytene spermatocytes (PS), but not late pachytene spermatocytes (LP). No nuclear staining was seen in mutant testis sections, although reddish cytoplasmic background is present. Identification of cell types was judged in part by estimating the epithelial stage of the tubules as described [67]. (G) TRIP13 localization in surface-spread spermatocytes. Preparations were immunolabeled with anti-SYCP3 (S) and TRIP13 (T). Both individual and merged images are shown for leptotene (Lep), zygotene (Zyg), and pachytene (Pac) spermatocytes. Nuclear staining was absent in the mutant.

The mammalian ortholog of *PCH2, Trip13 (*thyroid hormone receptor interacting protein 13), encodes a protein with extensive amino acid homology in regions alignable to the yeast and worm orthologs (Figure S1) [12]). Interestingly, phylogenetic analysis of TRIP13/Pch2p shows that the mammalian protein clusters more closely to plants than it does to the evolutionarily more closely related worms and flies (Figure 1A; see Discussion). Semi-quantitative reverse-transcriptase PCR (RT-PCR) analysis showed *Trip13* mRNA to be expressed in a variety of embryonic and adult tissues, including testis (Figure 1B), consistent with mouse and human EST data summarized in Unigene (http://www.ncbi.nlm.nih.gov/UniGene). It is also highly expressed in human and mouse oocytes [17].

### Generation of *Trip13* Mutant Mice

**Figure 2:**
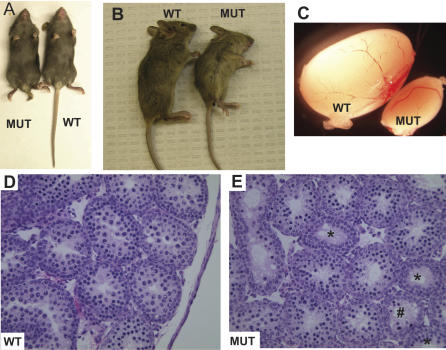
Developmental Phenotypes of *Trip13* Mutant Mice (A) Shown are 21-d-old littermates. Note the shortened tail in the mutant, but overall similar body size. (B) Shown are 23-d-old littermates. The mutant is smaller in this case, but the tail is not as truncated as the mouse in (A). (C) Wild-type (WT) and homozygous *Trip13* mutant (MUT) testes. (D) and (E) are cross sections through 17.5-d-old heterozygous (“WT”) and homozygous mutant Trip13 testes, respectively. Whereas the tubules in WT show coordinated spermatogenesis with pachytene spermatocytes present in all tubules (proximal to the lumen), developmental progression in the mutant is not synchronized between tubules. Some tubules have no pachytene spermatocyes (asterisks), while in others, development is somewhat disorganized (#).

To explore the function of TRIP13 in mammals, we generated mice with a gene trap-disrupted allele, *Trip13^RRB047^* (Figure 1C; abbreviated as *Trip13^Gt^*). Heterozygotes were normal in all respects, but homozygotes were present at ∼2/3 the expected ratio from intercrosses between heterozygotes (91 *Trip13* ^+/+^, 183 *Trip13^Gt/+^*, and 61 *Trip13^Gt/Gt^*). Since >90% of prewean mice that died were mutant homozygotes, this discrepancy is apparently due to a partially penetrant lethality. Most surviving *Trip13^Gt/Gt^* animals were grossly normal. However, homozygotes that were semi-congenic (N4) on the C57BL/6J strain were often markedly smaller and/or had kinked or shorter tails (Figure 2A and 2B).

RT-PCR analysis of *Trip13^Gt^* expression (Figure 1D) revealed a low level of normally spliced transcripts in testes of homozygotes that is presumably a consequence of incomplete usage of the gene trap's splice acceptor. Western blot analysis, using a polyclonal antibody raised against a peptide encoded by exon 3, revealed multiple species in wild-type and heterozygous testes, one of which corresponds to the expected size of 48 kDa (Figure 1E). This and three other species were undetectable in homozygous mutant testes, but a reduced amount of an intense ∼38 kDa smaller band was present. It is not clear if this corresponds to TRIP13. The greatly decreased *Trip13* mRNA and predicted correct-length protein in mutants indicate that the *Trip13^RRB047^* allele is severely hypomorphic.

To determine the germ cell types in which TRIP13 is expressed, and to assess possible expression in the mutant by means other than Western analysis, testis sections were immunolabeled for TRIP13 using a polyclonal chicken antipeptide antibody (see Materials and Methods). The most intensely labeled cells in control testes were Type B spermatogonia and leptotene spermatocytes (Figure 1F). Zygotene/pachytene spermatocytes stained less strongly, and there was no detectable staining in late pachytene spermatocytes. TRIP13 appeared to be nuclear localized. There was no such staining of nuclei in mutant seminiferous tubules (Figure 1F). To further assess the nuclear localization, TRIP13 was used to probe meiotic chromosomes prepared by surface spreading of spermatocyte nuclei. In wild type, there was diffuse nuclear staining, and no evidence of concentration on SC cores (marked by the axial element protein SYCP3) at any meiotic substage (Figure 1G). TRIP13 signal was noticeably absent in mutant meiotic nuclei.

### Infertility Due to Meiotic Disruption in TRIP13-Deficient Meiocytes

**Figure 3:**
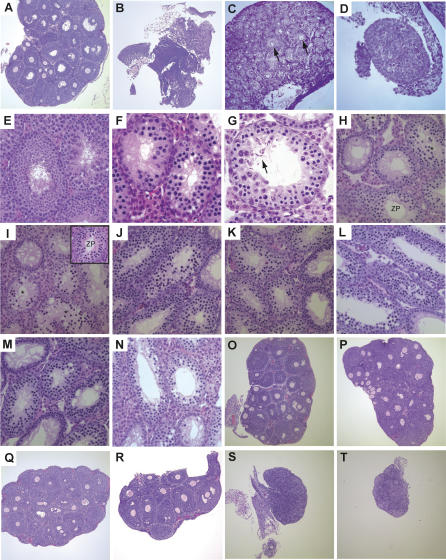
Histology of Mutant Gonads All are hematoxylin/eosin-stained paraffin sections. Testes are from 6-wk-old males, except as indicated below. (A) Wild-type 25-d-old ovary. (B) *Trip13^Gt^* ^/Gt^ 25-d-old ovary, showing dysgenesis from an absence of oocytes. (C) *Trip13^Gt^/+* 2-d-old control ovary. Arrows point to oocytes in newly forming follicles. (D) *Trip13^Gt/Gt^* 2-d-old ovary, dysgenic due to lack of oocytes. Magnification is the same as its littermate in “C.” (E) Wild-type testis. (F) *Trip13^Gt/Gt^* testis with uniform pachytene arrest. (G) *Trip13^Gt/Gt^* 3-mo-old testis with some postmeiotic spermatids (arrows). (H) *Spo11^−/-^* testis. A tubule with spermatocytes at leptotene/zygotene transition is labeled ZP, and tubules with apoptotic spermatocytes are marked with an asterisk. The specimen was taken from a littermate of that in (I). (I) *Spo11* ^−*/*−^ *Trip13^Gt/Gt^* testis. Labeling is the same as in (H). The inset contains a tubule with leptotene-zygotene spermatocytes. (J) *Mei1* ^−*/*−^ *Trip13^Gt^*/+ testis. The specimen was taken from a littermate of that in (K). (K) *Mei1* ^−*/*−^ *Trip13^Gt/Gt^* testis. (L) *Rec8^Mei8^*/*Rec8^Mei8^ Trip13^Gt^*/+ testis. The *Rec8^Mei8^* allele was described [39]. The specimen was taken from a littermate of that in (M). (M) *Rec8^Mei8^*/*Rec8^Mei8^ Trip13^Gt/Gt^* testis. (N) *Dmc1* ^−*/*−^ *Trip13^Gt/Gt^* testis. (O) *Spo11* ^−*/*−^ *Trip13^Gt^*/+ 25-d-old ovary. The specimen was taken from a littermate of that in (P). (P) *Spo11* ^−*/*−^ *Trip13^Gt/Gt^* 25-d-old ovary. (Q) *Mei1* ^−*/*−^ *Trip13^Gt^*/+ 25-d-old ovary. The specimen was taken from a littermate of that in (R). (R) *Mei1* ^−*/*−^ *Trip13^Gt/Gt^* 25-d-old ovary. (S) *Rec8^Mei8^*/*Rec8^Mei8^ Trip13^Gt^*/+ 25-d-old ovary. The specimen was taken from a littermate of that in (T). (T) *Rec8^Mei8^*/*Rec8^Mei8^ Trip13^Gt/Gt^* 25-d-old ovary.

Homozygotes of both sexes had small gonads (Figure 2C; see below) and were invariably sterile. Ovaries of adult *Trip13^Gt/Gt^* females were severely dysmorphic and had few or no follicles (Figure 3A and 3B). The majority of oocyte loss occurred in late embryogenesis or early in postnatal development, since 2 d postpartum ovaries were markedly smaller than those of control littermates, and were lacking oocytes or newly forming follicles (Figure 3C and 3D). Thus, oocytes failed to progress to the dictyate (resting) phase. Since we observed oocytes with pachytene stage chromosomes in 17.5 d *Trip13^Gt/Gt^* embryonic ovaries (unpublished data), this indicates that oocytes were eliminated somewhere between pachynema and dictyate.

Histological sections of mutant testes revealed a lack of postmeiotic cell types that are characteristic of wild-type seminiferous tubules (Figure 3E). The most developmentally advanced seminiferous tubules contained adluminal spermatocytes with condensed chromatin characteristic of pachynema (Figure 3F). The absence of coordinated spermatogenic progression beyond this stage is indicative of a pachytene arrest. This was revealed more clearly by chromosome analysis (see below). Some sections of adult seminiferous tubules contained postmeiotic spermatids (Figure 3G), although we saw no motile epididymal sperm. These drastic meiotic defects stand in contrast to yeast and *C. elegans,* in which deletion of *Pch2* alone has minor effects on spore/gamete development [2,8].

### TRIP13-Deficient Meiocytes Undergo Homologous Chromosome Synapsis Despite the Presence of Unrepaired DSBs in Pachynema

**Figure 4:**
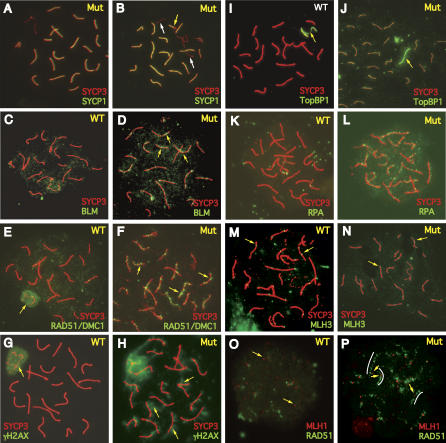
Immunohistochemical Analysis of Pachytene Spermatocyte Chromosomes Surface-spread chromosomes were immunolabeled with the indicated antibodies and fluorophores. As indicated in the upper right of each panel, cells were from wild type (WT, either +/+ or *Trip13^Gt/+^*) or *Trip13^Gt/Gt^* (Mut). There were no differences seen between heterozygotes and +/+ spermatocytes. (A) A mutant pachytene nucleus with full synapsis. Areas of SYCP1/SYCP3 colabeling are yellow. (B–E) Spermatocytes nucleus from 17.5 d postpartum mutant. Asynapsed chromosomes or regions of chromosomes are indicated by white and yellow arrows, respectively. Unlike the normal distribution in wild-type pachytene spermatocytes (C), BLM foci are present on synapsed pachytene chromosomes in the mutant (D). RAD51 foci, which are abundant earlier in prophase, disappear from autosomes in wild-type pachytene nuclei (E) and the bulk of staining is over the XY body (arrow). (F) RAD51 persists on the synapsed mutant chromosomes (arrows). (G) H2AX phosphorylation is restricted to the XY body in WT. (H) In addition to a large area of γH2AX staining (arrow) over the XY body, there is extensive autosomal H2AX phosphorylation (arrows). (I, J) Note that in wild-type pachytene spermatocytes, TOPBP1 is present only over the XY body (yellow arrow). In the mutant (J), an arrow denotes one area of intensive staining that may be over the sex chromosomes, but many other chromosome cores are positively stained. (K, L) RPA persists along synapsed cores in the mutant, not WT. (M, N) Arrows indicate examples of MLH3 foci on SCs. (O) In WT late pachytene spermatocytes, RAD51 is present only at background levels. (P) As in (F), extensive RAD51 staining delineates SCs in mutant pachytene nuclei (indicated by white arcs). MLH1 foci colocalize with these tracts (arrows) at the typical 1–2 foci per chromosome as in (M).

To better characterize the degree of meiotic progression in *Trip13^Gt/Gt^* spermatocytes, we immunostained chromosome spreads for SYCP3 and SYCP1, components of the axial/lateral elements and transverse filaments, respectively, of the synaptonemal complex (SC). Pachytene spermatocyte nuclei from postpubertal mutant testes could assemble normal SC cores and exhibited full synapsis of chromosomes as judged by colabeling of SYCP1 and SYCP3 along the full lengths of all autosomes (Figure 4A). Additionally, the X and Y chromosomes were normally synapsed at their pseudoautosomal region. More prepubertal (17.5 d postpartum) mutant spermatocytes contained asynaptic or terminally asynapsed chromosomes than age-matched controls (62.5% versus 25%, respectively; Figure 4B). We attribute this to a delay in the first wave of postnatal spermatogenesis (Figure 2D and 2E), likely related to systemic developmental retardation (Figure 2A and 2B). Nevertheless, since *Trip13^Gt/Gt^* spermatocytes progress to pachynema with no gross SC abnormalities, and oocytes were eliminated soon after birth (a characteristic of DNA repair mutants [13]), this suggested that unrepaired DSBs are responsible for eventual meiotic arrest and elimination.

To elucidate the cause of meiotic arrest, we analyzed meiotic chromosomes with a variety of markers that are diagnostic of recombination and synapsis. Recombination in *Trip13^Gt/Gt^* spermatocytes appeared to initiate normally as judged by the presence of γH2AX in leptonema (Figure S2A and S2B), which reflects the presence of meiotically induced DSBs [18]. RAD51 and/or DMC1, components of early recombination nodules (ERNs), was also present as abundant foci in *Trip13^Gt/Gt^* zygotene spermatocytes (unpublished data; the anti-RAD51 antibody cross-reacts with DMC1), indicating that recombinational repair of DSBs is initiated. The cohesin complex, which is essential for completion and/or maintenance of synaptic associations, appeared to assemble normally as judged by immunolabeling for the meiosis-specific cohesins STAG3 (Figure S2C and S2D) and REC8 (unpublished data). Because yeast PCH2 localizes to telomeres in a Sir3p-dependent manner, we tested for possible telomere defects by immunolabeling for TRF2, a component of a protein complex that plays an essential role in telomere protection [19]. It was localized to telomeres of both fully synapsed and telomerically asynaptic mutant chromosomes (Figure S2E and S2F).

Defects in DSB repair became apparent in pachynema upon probing of mutant spermatocyte nuclei with antibodies against molecules involved in various stages of recombination. In >99% of *Trip13^Gt/Gt^* chromosome spreads, BLM helicase (Figure 4C and 4D), RAD51/DMC1 (Figure 4E and 4F), γH2AX (Figure 4G and 4H), and TOPBP1 (Figure 4I and 4J) all persisted abnormally on synapsed chromosomes. For RAD51/DMC1, mutant pachytene spermatocytes contained 138 ± 6 foci (compared to 11 ± 3 foci in wild type, most of which were on the XY body), down from 218 ± 13 in zygonema (compared to 220 ± 13 foci in wild type). TOPBP1 is a DNA damage–checkpoint protein involved in ATM protein–dependent activation of ATR protein [20,21]. It binds sites of DSBs and unsynapsed regions of meiotic chromosomes [22,23]. BLM has been reported to colocalize with markers (RPA and MSH4) of recombination at sites distinct from those that become resolved as crossovers (CO) [24]. We therefore assessed the distribution of RPA, the ssDNA binding protein, which is normally present at focal sites of synapsing meiotic chromosomes before disappearing in mid-pachynema [25]. It is thought to bind D-loops of recombination intermediates [26]. RPA also persisted on pachytene mutant chromosomes (Figure 4K and 4L). These data indicate that unrepaired DSBs, or unresolved recombination intermediates, remain in pachynema and activate a DNA damage checkpoint system.

It should be noted that chromosomes affected by meiotic sex chromosome inactivation (MSCI) and meiotic silencing of unpaired chromatin (MSUC) are heavily stained by antibodies for several DSB repair-associated molecules, including γH2AX. H2AX phosphorylation due to MSCI and MSUC is conducted by ATR, not ATM [27–29]. Since mutant chromosomes are fully synapsed, and MSUC is known to occur only as a result of asynapsis, the decoration of *Trip13^Gt/Gt^* chromosomes with DNA repair markers is probably attributable to incomplete DNA repair rather than transcriptional silencing.

Consistent with the presence of rare (<1%) *Trip13^Gt/Gt^* pachytene spermatocytes devoid of persistent DNA repair markers, and testis histology showing some degree of postmeiotic progression (Figure 3G), we observed both diplotene nuclei that lacked autosomal RAD51/DMC1 and γH2AX (Figure S3A–S3D), and also metaphase I spreads with 20 bivalents (Figure S3E–S3F). Since *Trip13^Gt^* may not be a complete null, these diplotene and metaphase I spermatocytes might arise by virtue of having sufficient wild-type TRIP13.

### CO-Associated Markers Appear Normally in the Absence of TRIP13

The persistence of BLM on *Trip13^Gt/Gt^* spermatocyte chromosomes suggests that at least a subset of the unrepaired DSBs correspond to sites of defective NCO recombinational repair. To assess whether CO recombination occurs in the mutant, we examined the distribution of the mismatch repair proteins MLH1 and MLH3, which are normally detectable as foci in mid-late pachynema and mark the locations of chiasmata [30,31]. Remarkably, MLH1/3 foci were formed; we observed 1–2 foci/chromosome as in wild type and at typical overall levels (MLH3 = 23 ± 2, *N* = 10; [30,32]) on mid-late pachytene chromosomes (Figure 4M and 4N; MLH1 not shown). Since <1% of *Trip13^Gt/Gt^* pachytene nuclei had normal repair (as judged by absence of persistent DSB repair markers; see above), but most of the pachytene nuclei had MLH1/3 foci, it was unlikely that the MLH1/3 foci formed only on chromosomes with fully repaired DSBs. To test this directly, we conducted double staining for MLH1 and RAD51/DMC1. MLH1 foci were present on chromosomes that also contained numerous RAD51/DMC1 foci (Figure 4O and 4P).

To assess whether these MLH1/3 foci in *Trip13^Gt/Gt^* pachytene spermatocytes represent CO events completed to a point where they could maintain interhomolog attachments though the end of prophase I, we treated testicular cells from 17.5–20.5-d-old control (+/+), *Trip13^Gt/Gt^,* and *Dmc1* ^−*/*−^ mice with the protein phosphatase inhibitor okadaic acid (OA), a chemical that induces degradation of the SC, chromosome condensation, and premature progression to metaphase I [33]. Fifteen metaphase spreads were identified for each genotype. Whereas all of the *Dmc1* ^−*/*−^ spreads had ∼35 or more condensed chromosomes, all of the +/+ and *Trip13^Gt/Gt^* spreads had 20–25, suggesting that the MLH1/3 foci in *Trip13^Gt/Gt^* pachytene spermatocytes represent sites of completed, or near-completed, COs. Because the preparations were made from whole testes, it is possible that the univalent-containing metaphases from *Dmc1* ^−*/*−^ mice were from spermatogonia, not spermatocytes.

### TRIP13 Deficiency Does Not Alleviate Meiotic Arrest Phenotypes of Mutants Defective in Synapsis

**Figure 5:**
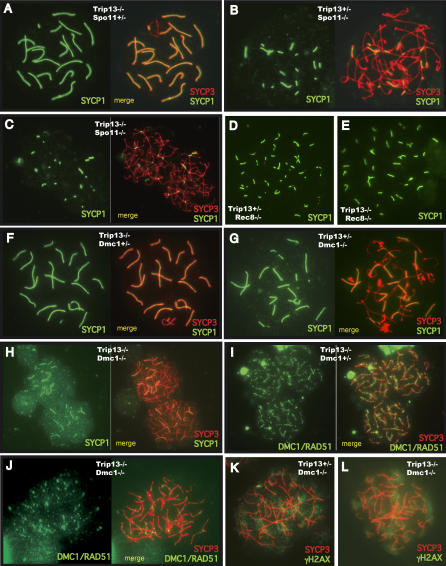
Immunocytological Analysis of *Trip13* Compound Mutants Surface-spread chromosomes were immunolabeled with the indicated antibodies and fluorophores. Genotypes are indicated, as are those panels in which dual staining patterns are merged. Note that (H) and (I) are at lower magnification to show multiple nuclei.

To determine if TRIP13 deficiency prevents apoptosis triggered by asynapsis as in *C. elegans,* we analyzed mice that were doubly mutant for *Spo11* and *Trip13*. SPO11 is a transesterase that is essential for the creation of genetically programmed DSB during leptonema of many organisms, including mice [18]. In *C. elegans, spo-11* mutant gametes have extensive asynapsis, which triggers PCH-2 dependent apoptosis in pachynema [2]. In mice, *Spo11* ^−*/*−^ spermatocytes are severely defective in homologous chromosome synapsis [34,35], and arrest with chromosomes in a state characteristic of the zygotene/pachytene transition (Figure 3H). Spermatocytes in *Trip13^Gt/Gt^ Spo11* ^−*/*−^ testes progressed maximally to that point before undergoing death (Figs 3I), well before the spindle checkpoint that eliminates achiasmate spermatocytes [36]. There was no evidence of metaphase I spermatocytes or postmeiotic spermatids in these testes, unlike those seen in *Trip13* single mutants (Figure 3G). In contrast to the complete synapsis in *Trip13^Gt/Gt^* pachytene spermatocytes (Figure 5A), in which SPO11 is available in leptonema to initiate (via DSB induction, Figure S2A and S2B) a recombination-driven homolog search, chromosome synapsis in doubly mutant spermatocytes was highly disrupted as in *Spo11* single mutants (Figure 5B and 5C). Identical studies were performed with mice deficient for *Mei1,* a vertebrate-specific gene also required for DSB formation and chromosome synapsis [37], with similar results (Figure 3J and 3K; immunocytology not shown).

In yeast, deletion of PCH2 alleviates the pachytene arrest caused by asynaptic mutants *zip1* and *zip2* [8]. Although mouse SYCP1 might be a functional equivalent of Zip1p, because *Sycp1* mutant spermatocytes arrest at approximately the same point as *Trip13* mutants, there would be no opportunity to observe bypass of *Sycp1* ^−*/*−^. Since Zip2p is present at sites of axial associations, even in *zip1* mutants, it has been suggested that Zip2p promotes initiation of chromosome synapsis [38]. These observations raise the possibility that in yeast, Pch2p responds to synapsis polymerization rather than initiation. To test this, we performed epistasis analysis with a *Rec8* allele (*Rec8^Mei8^,* abbreviated as *Rec8* ^−^). Meiotic chromosomes of *Rec8* mutant spermatocytes undergo apparent homolog pairing and interhomolog synaptic initiation, but are defective in DSB repair and fail to maintain interhomolog synapsis [39,40]. Rather, sister chromatids appear to synapse and are bound by SYCP1 along their axes. *Rec8* mutants do not progress to diplonema or metaphase I. Double mutant analysis indicated that *Rec8* is epistatic to *Trip13*. As in the *Spo11* and *Mei1* experiments, histology of testes deficient for both REC8 and TRIP13 resembled the *Rec8* mutant, with no evidence of progression to metaphase I that occurs in *Trip13^Gt/Gt^* mice (Figure 3L and 3M). Immunocytological analysis of spread chromosomes showed a failure of homologous chromosome synapsis in both the *Rec8* ^−*/*−^ and *Rec8* ^−*/*−^ *Trip13^Gt/Gt^* spermatocytes, as previously reported for *Rec8* mutants (Figure 5D and 5E) [39,40].

Although subsequent reports indicate otherwise [10,12], deletion of *PCH2* in yeast was originally reported to alleviate meiotic arrest caused by deficiency for the meiosis-specific RecA homolog *DMC1* [8]. To investigate this relationship in mice, we constructed animals doubly mutant for *Trip13* and *Dmc1*. As in *Dmc1* ^−*/*−^ mice, in which spermatocytes undergo meiotic arrest from defective DSB repair and failed chromosome synapsis [16], spermatogenesis in *Dmc1* ^−*/*−^ *Trip13^Gt/Gt^* testes was uniformly arrested at the point where spermatocytes contained chromatin characteristic of zygonema/pachynema (Figure 3N). Immunocytological analysis indicated that both *Dmc1* ^−*/*−^ and *Dmc1* ^−*/*−^ *Trip13^Gt/Gt^* chromosomes had extensive asynapsis compared to *Trip13^Gt^* single mutants (Figure 5F–5H), and all had persistent RAD51/DMC1 foci and phosphorylated H2AX (γH2AX; Figure 5I–5L), confirming that *Dmc1* is epistatic to *Trip13*. Doubly mutant females had residual ovaries, phenocopying *Dmc1* ^−*/*−^ and *Trip13^Gt/Gt^* single mutants (unpublished data).

### Meiotic Defects in *Trip13^Gt/Gt^* Oocytes Are DSB-Dependent

Epistasis analysis of females was insightful with respect to the cause of arrest in *Trip13* mutants. Both *Mei1* ^−*/*−^/*Trip13^Gt/Gt^* and *Spo11* ^−*/*−^/*Trip13^Gt/Gt^* females had ovaries with numerous follicles, identical to *Mei1* and *Spo11* single mutants (Figure 3O–3R). Thus, *Spo11* and *Mei1* are epistatic to *Trip13* in oogenesis, just as they are to *Dmc1* [13,41]. This demonstrates that oocyte loss in *Trip13^Gt/Gt^* females is dependent on DSB formation. In conjunction with the immunohistochemical data, these data provide strong evidence that meiotic arrest in *Trip13* mutant mice is due to defects in DSB repair. As expected, ovaries of *Rec8 Trip13* double mutants were devoid of oocytes as were those from either single mutant (Figure 3B, 3S, and 3T).

## Discussion

Genetic experiments in S. cerevisiae provided evidence that the pachytene checkpoint monitors and responds to recombinational DSB repair and synapsis independently. Wu and Burgess concluded that the repair checkpoint is *RAD17*-*SAE2* dependent, while the synapsis checkpoint is *PCH2*-*ZIP1* dependent [12]. Of these four genes, *SAE2* and *ZIP1* do not have clear mammalian orthologs (although SYCP1 may be a functional ortholog of ZIP1), and mutation of the mouse *RAD17* ortholog, *Rad1,* presumably causes embryonic lethality [42]. Thus, mutational analysis of mouse *Pch2 (Trip13),* which is also critical for the synapsis checkpoint in C. elegans [2], was the best remaining option to evaluate potential functional conservation in mammalian meiotic checkpoint control.

Our results demonstrate that in mice, the primary meiotic function of TRIP13 is in recombination itself. We found no evidence that it is involved in pachytene checkpoint control. Our data suggest that while recombination events destined to be resolved as COs can proceed normally in *Trip13* mutants, DSBs that enter the NCO repair pathway are incompletely resolved or processed inefficiently. This hypothesis is compatible with current knowledge of meiotic recombination pathways. In *S. cerevisiae,* CO and NCO pathways are distinct [43]; they have different recombination intermediates, and are dependent upon different proteins [44,45]. Mice also appear to have independent CO versus NCO recombination pathways [46]. As in yeast, both require SPO11-induced breaks, but only the CO pathway requires MLH1. Both types of recombinant products are formed by mid-late pachynema. Another possibility is that the recombination defects are a result of defective intersister recombination. However, this type of DSB repair is suppressed in meiotic cells. Ablation of *RAD54,* which mediates intersister recombination in yeast, does not significantly disrupt meiosis in either yeast or mice [47,48]. Interestingly, RAD54-deficient spermatocytes display abnormal persistence of RAD51 foci on pachytene chromosomes, similar to those in TRIP13 mice, but there are no deleterious effects on meiotic progression or fertility [49].

Data from budding yeast also indicate that Pch2p functions in recombination. Deletion of *PCH2* delays meiotic progression by ∼2 h in SK1 yeast, and causes a minor decrease in ascus formation [50]. DSBs persist >2 h longer in *pch2*Δ yeast than in wild type, and hyperresection of DSBs in *dmc1*Δ *pch2*Δ double mutants is lower than in *dmc1*Δ cells [10]. Additionally, it was reported that *pch2*Δ yeast had a meiosis I delay dependent on the *RAD17–SAE2* checkpoint that monitors recombination intermediates [12]. However, the exact role of TRIP13 (or Pch2) in recombination is unclear. Because synapsis occurs in TRIP13-deficient spermatocytes and is dependent on DSB formation (activity of SPO11 and MEI1), we suggest that TRIP13 functions after homology recognition and strand exchange, and that recombination events entering the CO repair pathway are either completed or nearly so (because OA treated resulted in bivalent chromosomes). One possibility for TRIP13′s role in recombination is that it is directly involved in a step specific to resolution of NCO recombination intermediates. Another possibility is that TRIP13 is required for disassembly of NCO recombinational repair complexes [51] containing those proteins that persist abnormally on *Trip13^Gt/Gt^* pachytene chromosomes. Notably, TRIP13 has two putative ATPase domains, a signature of AAA-ATPase ClpA/B chaperones that perform protein or protein/DNA complex disassembly [52]. These potential recombination roles might not be limited to meiosis, since *Trip13* is widely transcribed and the mutant animals exhibited developmental defects. Finally, TRIP13 might play an indirect role, such as providing a “licensing” signal for the resolution of NCO intermediates and completion of meiosis.

Regarding the cause of cell death in *Trip13* mutants, our data indicate that this is triggered by defective DSB repair rather than asynapsis. We base this conclusion on two observations: (1) oocyte elimination is dependent upon DSB formation and (2) synapsis is normal in spermatocytes of adult testes. Indeed, this mutant is unique in that recombination defects occur in the absence of asynapsis (e.g., as in *Dmc1* knockouts). Thus, the *Trip13* mutant provides the first evidence that unrepaired DNA damage alone can trigger the mammalian pachytene checkpoint response. Furthermore, our results allow us to conclude that oocytes and spermatocytes share a similar, if not identical, DNA damage pachytene checkpoint that is decoupled from a synapsis checkpoint.

Interestingly, we found that OA treatment of *Trip13^Gt/Gt^* spermatocytes could propel them into MI, despite a report that the same did not occur when wild-type pachytene spermatocytes were treated with the DSB-inducing agents gamma radiation or etoposide [53]. It is possible that the nascent induction of DSBs in pachynema evokes a checkpoint response that cannot be bypassed by OA, whereas the post-strand invasion lesions in TRIP13-deficient spermatocytes do not.

TRIP13 was originally discovered to be an interactor with rat thyroid receptor beta (THRB; [54]), but the relationship between THRB and TRIP13 in meiosis is unknown. Interestingly, we observed that THRB is distributed diffusely throughout wild-type spermatocyte nuclei but is excluded from the XY (sex) body (unpublished observations), a compartmentalized nuclear domain beginning in pachynema, in which the sex chromosomes become heterochromatinized and transcriptionally silenced in the process of MSCI [55]. However, the XY body appeared intact in most mutant spermatocytes upon probing with several markers of XY heterochromatinization (unpublished observations). Considering that THRB knockout mice are viable and fertile [56], the functional relationship between TRIP13 and its receptor THRB in meiosis is unclear.

Given the high similarity of PCH2 orthologs throughout the eukaryotic world, one or more essential functions of this protein must be conserved. Since TRIP13 does not exhibit checkpoint function in mice, we surmise that the TRIP13/PCH2 ancestral protein had a function in recombination that persists to the present. Notably, A. thaliana does not appear to have a meiotic checkpoint activity that eliminates mutant meiocytes in a manner analogous to organisms such as mice, budding yeast, and female *Drosophila* [11,57], and mammalian TRIP13 is more similar to *Arabidopsis* PCH2 than the fly or worm proteins (Figures 1A and S1). The unusual relatedness between mammalian and plant PCH2 may therefore be attributable to both the presence of a common conserved function (namely recombination, although the role of PCH2 in plants has yet to be determined), and the absence of checkpoint function. Nevertheless, the evolutionary relationships between animals, fungi, and plants (which are discordant with PCH2 sequence phylogeny) do not allow parsimonious models addressing the points in time that checkpoint functions in PCH2 were gained or lost. It is possible that its checkpoint function evolved independently in worms and budding yeast. The picture will become clearer as the function of PCH2 in other organisms is elucidated.

The nature of the synapsis checkpoint in male mice remains unidentified. One possible candidate is *Dot1* (*PCH1* in yeast), a histone methyltransferase silencing factor that is required for pachytene arrest of *zip1* and *dmc1* mutants in yeast [58], and for preventing RAD54-mediated recombinational DSB repair between sister chromatids. However, *DOT1* acts upstream of *PCH2*. Given that TRIP13 doesn't have checkpoint function in mice, a potential role for mammalian DOT1 in the pachytene checkpoint is dubious but awaits investigation. Recently, it was shown that the TRP53 homolog TRP63 is required for DNA damage–induced death of dictyate-stage primordial oocytes, leading to the suggestion that it is involved in monitoring genome integrity [59]. However, this activity occurs subsequent to a pachytene checkpoint. As alluded to earlier, a complicating problem for studying potential meiotic checkpoint genes in mice is that as in yeast, such genes often have mitotic functions (such as RAD24 [7]), and their ablation can cause lethality [42]. Unless mammalian pachytene checkpoint components have orthologs with similar functions in organisms such as yeast, their identities are likely to remain elusive.

## Materials and Methods

### PCR analysis of *Trip13* cDNA.

*Trip13* was amplified from samples of Clontech's Mouse Multiple Tissue cDNA Panel I (http://www.clontech.com), using the following primers: 5′-GCACCATTGCACTTCACATC-3′ (TRP3-6F) and 5′-TGACCATCAGACTGTCGAGC-3′ (TRP3-6R). These primers correspond to exons 3 and 6, respectively, and amplify a 330-bp cDNA product. The cDNAs in this panel are equalized to allow quantitative analysis by RT-PCR.

### Generation of *Trip13*-deficient mice.

The mouse embryonic stem cell line RRB047 (strain 129/Ola) containing a gene trap insertion in *Trip13* was obtained from BayGenomics (http://www.baygenomics.ucsf.edu/). The gene-trapping vector used to create this line, pGT1lxf, was designed to create an in-frame fusion between the 5′ exons of the trapped gene and a reporter, *βgeo* (a fusion of β-galactosidase and neomycin phosphotransferase II). The gene-trapped locus creates a fusion transcript containing exons 1–3 of *Trip13* and *βgeo*. To identify the exact insertion site within intron 3, PCR was performed using one primer within the gene trap vector, and the other primer at various positions in intron 3 pointing towards the 3′ end of the gene. Product from a productive reaction was sequenced, revealing that the insertion site was 445 bp into intron 3.

### Genotyping of mice.

Three primers were used to distinguish wild-type and mutant alleles of *Trip13:* primer 1, 5′-CGTCGCTCCATTGCTTTGTGC-3′; primer 2, 5′-AGTAGTGGTACACTGTATTTTTGCTTTCATTGA-3′; and primer 3, 5′-GTAGATCCCGGCGCTCTTACCAA-3′. Primers 1 and 2 are located upstream and downstream, respectively, of the gene trap insertion within the intron 3. Primer 3 corresponds to pGTlxf sequence. Primers 1 and 2 amplify a 700-bp band from a wild-type allele; primers 1 and 3 amplify a 540-bp fragment from a mutant allele. Separate reactions were used to assay the presence or absence of each amplicon from a DNA sample. The cycling conditions were: 94 °C 2 min; 35 cycles of 94 °C 30 s, 57 °C 45 s, and 72°C 50 s; and 72 °C 2 min.

### RT-PCR.

Total RNA was isolated from adult testes with the RNeasy Mini Kit (Qiagen, http://www.qiagen.com), and 4.0 μg was oligo dT–primed and reverse-transcribed with Superscript II (Stratagene, http://www.stratagene.com). The entire *Trip13* protein-coding sequence was amplified with primers 5′-ATGGACGAGGCGGTG-3′ and 5′-TCAAACATAAGCTGAAAGTT-3′. The cycling conditions were: 94 °C 2 min; 94 °C 30s, 55 °C 45 s, and 72 °C 80 s for 35 cycles; and 72 °C 2 min. The primers for amplifying the *Med31* coding sequence as control were : 5′-ATGGCCGCGGCCGTCGCTATGG-3′ and 5′-TCATTTCCCTGCTGTGTTATTCTGCTGCTGCTGC-3′. The cycling conditions were: 94 °C 2 min; 94 °C 30 s, 55 °C 30 s, and 72 °C 35 s for 35 cycles; and 72 °C 2 min.

### Development and purification of chicken antibodies.

A peptide corresponding to amino acids 25–40 of TRIP13, VLQRSGSTAKKEDIK, was conjugated to KLH and used to immunize chickens (done by Sigma Genosys, http://www.sigmaaldrich.com). Polyclonal IgY was isolated from eggs with the Eggcellent Chicken IgY Purification kit (Pierce, http://www.piercenet.com). IgY antibodies were then affinity purified using the immunizing synthetic peptide.

### Western blotting.

50 μg of testis extract in RIPA buffer was separated by 8% SDS-PAGE and electrotransferred onto a Pure Nitrocellulose membrane (Bio-Rad, http://www.biorad.com). The membrane was incubated with a polyclonal rabbit anti-human TRIP13 antibody (18-003-42687; Genway, http://www.genwaybio.com). According to the manufacturer, the immunogen was a synthetic peptide embedded in sequence we deduced to correspond to exon 3. Binding was detected by chemiluminescence ECL kit (Pierce) using a rabbit anti-chicken IgG horseradish peroxidase conjugate (Pierce).

### Histological analyses.

Testes or ovaries were fixed in Bouin's, embedded in paraffin, sectioned at 6 μm, and stained by hematoxylin and eosin. Antigen retrieval for immunohistochemistry of testis sections was as described [60]. Oocyte and follicle numbers were counted as described [61]. Only follicles containing an oocyte with a clearly visible nucleus were scored.

### Immunocytochemistry.

Immunolabeling of surface-spread spermatocytes and oocytes was performed as described [39,62]. To reach conclusions on the pattern of staining for various proteins, 30 (unless otherwise indicated) well-spread nuclei of particular meiotic stages were first identified under the fluorescent microscope on the basis of SYCP3 or STAG3 staining, then imaged at both appropriate wavelengths to determine the pattern of second proteins with focal patterns such as RAD51 or RPA. Unless otherwise indicated, the panels shown in the figures were the exclusive or predominant patterns seen. The exception for this approach was in the case of staining for MLH1 or MLH3 plus RAD51 (in which case SYCP3 or STAG3 was not available to find chromosome cores). Nuclei in this situation were identified first by MLH1/3 foci clustering, then imaged for both fluorescent wavelengths.

Primary antibodies used in this study were as follows: mouse anti-SCP3 (1:500; Abcam, http://www.abcam.com); rabbit anti-SYCP1 (1:1,000; a gift from C. Heyting) [63]; rabbit anti-REC8 (1:100; a gift from C. Heyting); rabbit anti-RAD51 (1:250, this polyclonal antibody recognizes both RAD51 and DMC1; Oncogene Research Products, http://www.merckbiosciences.co.uk); rabbit anti-γH2AX (1:500; Upstate Biotechnology, http://www.upstate.com/); rabbit anti-STAG3 (1:1,000; a gift from R. Jessberger); rabbit anti-MLH3 (1:400; a gift from P. Cohen); mouse-anti-human MLH1 (1:50; BD Biosciences, http://www.bdbiosciences.com); rabbit-anti-TopBP1 (1:100; a gift from J. Chen) [22]; mouse-anti-ubiquityl-histone H2A (1:200; Upstate Biotechnology); rabbit-anti-TRF2 (1:500; a gift from T. de Lange); and rabbit-anti-BLM (1:50; a gift from R. Freire). All secondary antibodies conjugated with either Alexa Fluor 488 or 594 (Molecular Probes, http://probes.invitrogen.com/) were used at a dilution of 1:1,000. All images were taken with a 100× objective lens under immersion oil.

### Metaphase I spermatocyte spreads and OA treatment.

Metaphase fixed spermatocytes from 8-mo-old *Trip13^RRB047^* homozygotes, using 23-d-old wild-type mice as control, were prepared and stained with Giemsa as described [64].

For OA treatment, cells were exposed to 5 μM OA (Calbiochem, http://www.emdbiosciences.com) for 6 h at 32 °C in a humidified environment of 5% CO_2_ before spreading [65]. These preparations were stained with DAPI to visualize metaphase nuclei and chromosomes.

### Phylogenetic analyses.

TRIP13 orthologs were identified by BLASTP searches of Genbank and other sources providing gene models such as Ensembl. The selected orthologs can be found in Table S1. Amino acid alignments were done with Clustal W, using the default settings with and without removing the regions outside of the AAA-ATPase central domain. The trees were constructed by using the neighbor-joining method with Poisson correction. The reliability of internal branches was assessed by using 500 bootstrap replicates, and sites with gaps were ignored in this analysis. Neighbor-joining searches were conducted by using the computer program MEGA3 [66].

## Supporting Information

Figure S1 Depiction of Conserved Regions of Mouse TRIP13 and Its PCH2 Orthologs (75 KB PDF)

Figure S2 Surface-Spread Chromosomes Immunolabeled with the Indicated Antibodies and Fluorophores (947 KB PDF)

Figure S3 Trip13 Mutant Spermatocytes That Progress Beyond Pachynema Have Repaired DSBs and Form Bivalents at Metaphase I (1.5 MB PDF)

Table S1 Sources of TRIP13 Amino Acid Sequences Used to Construct the Phylogenetic Tree in Figure 1
(22 KB PDF)

## Abbreviations

CO: crossover
DSB: double strand break
MSCI: meiotic sex chromosome inactivation
MSUC: meiotic silencing of unpaired chromatin
NCO: noncrossover
OA: okadaic acid
RT-PCR: reverse-transcriptase PCR
SC: synaptonemal complex
