# Supplementary material for: Mouse Pachytene Checkpoint 2 (Trip13) Is Required for Completing Meiotic Recombination but Not Synapsis
Source: PLoS Genet. 2007 Aug 10;3(8):e130. doi: 10.1371/journal.pgen.0030130 (PMC1941754; doi:10.1371/journal.pgen.0030130)
Supplement: Supplementary file 1 [file pgen.0030130.sg001.pdf]

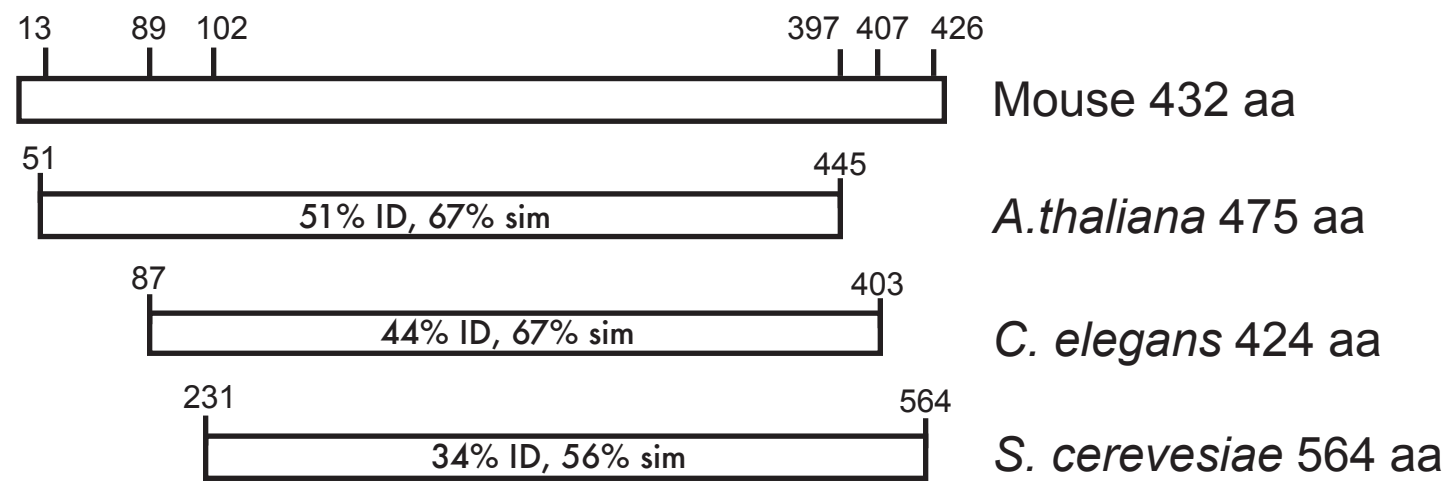

Fig. S1 - Depiction of conserved regions of mouse TRIP13 and its PCH2 orthologs.
