# Supplementary material for: Mouse Pachytene Checkpoint 2 (Trip13) Is Required for Completing Meiotic Recombination but Not Synapsis
Source: PLoS Genet. 2007 Aug 10;3(8):e130. doi: 10.1371/journal.pgen.0030130 (PMC1941754; doi:10.1371/journal.pgen.0030130)
Supplement: Supplementary file 2 [file pgen.0030130.sg002.pdf]

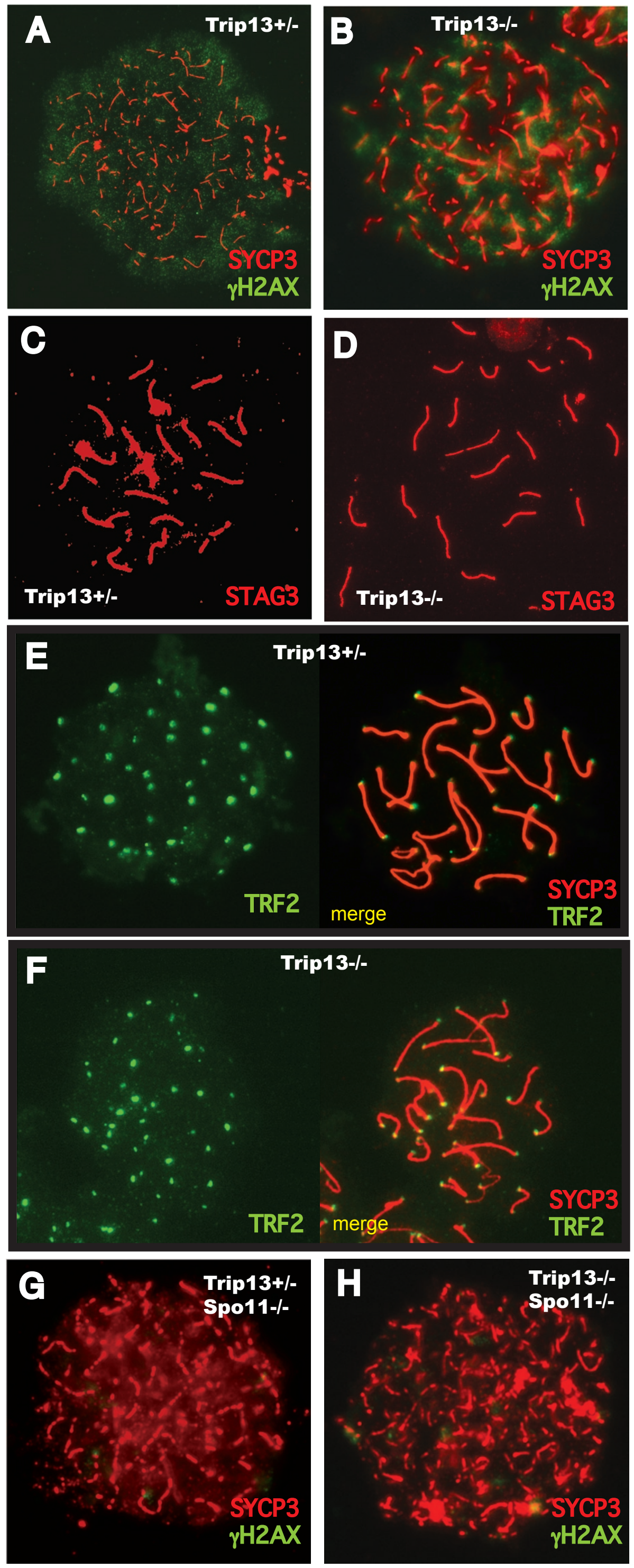

Figure S2. Surface-spread chromosomes were immunolabelled with the indicated antibodies and fluors. The nuclei in panels A, B, G and H are in leptonema, and the rest are in pachynema.
