# Supplementary material for: Mouse Pachytene Checkpoint 2 (Trip13) Is Required for Completing Meiotic Recombination but Not Synapsis
Source: PLoS Genet. 2007 Aug 10;3(8):e130. doi: 10.1371/journal.pgen.0030130 (PMC1941754; doi:10.1371/journal.pgen.0030130)
Supplement: Supplementary file 3 [file pgen.0030130.sg003.pdf]

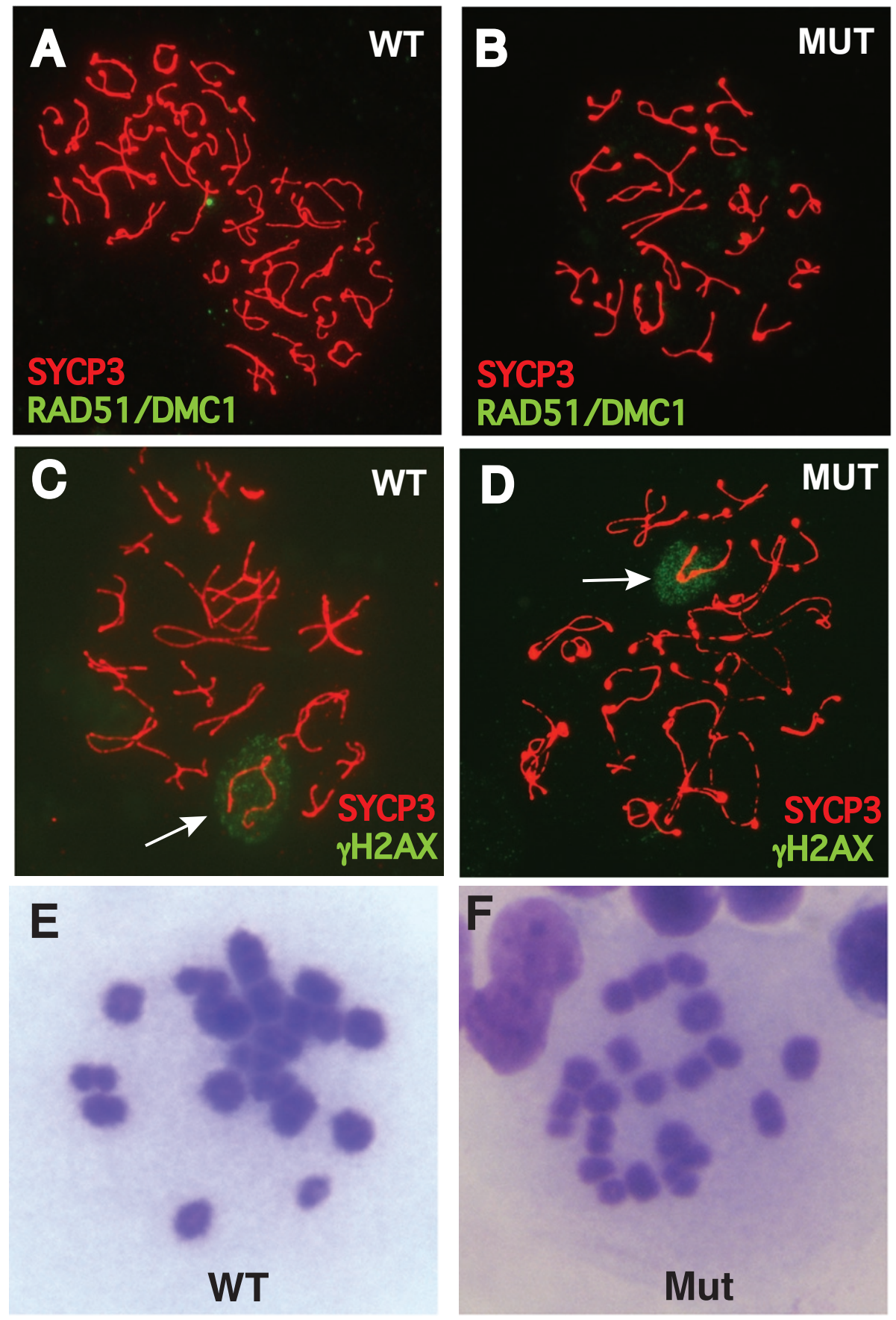

Figure S3 - Trip13 mutant spermatocytes that progress beyond pachynema have repaired DSBs and form bivalents at Metaphase I. (**A-D**) Diplotene stage control ("WT") and *Trip13*$^{Gt/Gt}$ ("MUT") spermatocytes labeled with indicated antibodies. Note the absence of pervasive RAD51/DMC1 binding and the XY body (arrows). (**E,F**) Metaphase I spreads show that mutant spermatocytes exhibit 20 bivalents as in normal cells, suggesting the presence of chiasmata to hold homologous chromosomes together.
