# Supplementary material for: Mouse Pachytene Checkpoint 2 (Trip13) Is Required for Completing Meiotic Recombination but Not Synapsis
Source: PLoS Genet. 2007 Aug 10;3(8):e130. doi: 10.1371/journal.pgen.0030130 (PMC1941754; doi:10.1371/journal.pgen.0030130)
Supplement: Supplementary file 4 [file pgen.0030130.st001.pdf]

Species	Protein/Gene ID	Gene Name
Bos taurus	ENSBTAG00000006972	Trip13
Caenorhabditis elegans	F10B5.5	Pch2
Canis familiaris	ENSCAFG00000010838	609426
Ciona savignyi	ENSCSAVG00000007117	novel Ensembl prediction
Danio rerio	ENSDARG00000025043	Trip13
Drosophila melanogaster	CG31453	CG31453
Gallus gallus	ENSGALG00000012508	420798
Homo sapiens	ENSG00000071539	Trip13
Macaca mulatta	ENSMMUG00000014740	Trip13
Monodelphis domestica	ENSMODG00000000818	Trip13
Rattus norvegicus	ENSRNOG00000015810	Trip13
Saccharomyces cerevisiae	YBR186W	Pch2
Takifugu rubripes	NEWSINFRUG00000136965	Novel Ensembl prediction
Tetraodon nigroviridis	GSTENG00034091001	GSTENG00034091001
Xenopus tropicalis	ENSXETG00000013105	Trip13
Mus musculus	ENSMUSG00000021569	Trip13
Caenorhabditis briggsae	BP:CBG00732	BP:CBP13887
Anopheles gambiae	ENSANGT00000011549.3	Q7PXQ3_ANOGA
Drosophila pseudoobscura	GA16260-PA	GA16260-PA
Oryza sativa	CAE05878 gi:38605777	OSJNBa0044K18.20
Arabidopsis thaliana	NP_194202	AT4G24710.1
Ashbya gossypii	NP_984603	AEL258W
Debaryomyces hansenii	XP_460723	DEHA0F09229g
Candida glabrata	XP_449627	CAGL0M06435g
Kluyveromyces lactis	XP_455025	unnamed protein product

Note: ID numbers prefixed by ENS originate from Ensembl.
